# A rare cause of echogenic kidneys with oligohydramnios in the fetus: report of two different cases

**DOI:** 10.1186/s12884-024-06861-w

**Published:** 2024-10-11

**Authors:** Tim Phetthong, Krit Achaloetvaranon, Sanpon Diawtipsukon

**Affiliations:** 1 Division of Medical Genetics, Department of Pediatrics, Phramongkutklao Hospital and Phramongkutklao College of Medicine, Bangkok, Thailand; 2 Department of Pathology, Phramongkutklao Hospital and Phramongkutklao College of Medicine, Bangkok, Thailand; 3 Department of Obstetrics and Gynecology, Chumphon Khet Udomsakdi Hospital and Clinical Medical Education Center, Chumphon, Thailand; 4 Division of Maternal-Fetal Medicine, Department of Obstetrics and Gynecology, Faculty of Medicine Ramathibodi Hospital, Mahidol University, Bangkok, Thailand

**Keywords:** Echogenic fetal kidney, Polycystic kidney disease, Glomerulocystic kidney disease, Autosomal recessive polycystic kidney disease

## Abstract

**Background:**

Prenatal ultrasound findings of fetal bilateral echogenic kidneys accompanied by oligohydramnios can be highly stressful for both pregnant women and physicians. The diversity of underlying causes makes it challenging to confirm a prenatal diagnosis, predict postnatal outcomes, and counsel regarding recurrence risks in future pregnancies.

**Case Presentation:**

We report two cases of abnormal fetal echogenic kidneys with oligohydramnios detected in the early third trimester. Autosomal recessive polycystic kidney disease (ARPKD), a rare genetic syndrome, was initially suspected in both cases. However, postnatal diagnoses differed: the first case was confirmed as glomerulocystic kidney disease (GCKD) through renal pathology, while the second case was diagnosed as ARPKD with a compound heterozygous likely pathogenic *PKHD1* mutation.

**Conclusion:**

Prenatal diagnosis of fetal echogenic kidneys with oligohydramnios should prioritize accurate diagnosis. Given the differences in the clinical spectrum, GCKD should be considered a differential diagnosis for this condition, particularly ARPKD. This study highlights the importance and benefits of molecular diagnosis and postnatal renal pathology for precise diagnosis and effective counseling.

**Supplementary Information:**

The online version contains supplementary material available at 10.1186/s12884-024-06861-w.

## Background

Fetal echogenic kidneys, identified by an abnormally bright appearance on ultrasound compared to the liver or spleen, can indicate underlying renal abnormalities and dysfunction [1, 2]. While echogenic kidneys may sometimes present as an isolated finding, their association with factors such as enlargement (greater than the 95th percentile), cystic changes, reduced amniotic fluid, or a family history of renal diseases increases the likelihood of significant renal issues in the fetus [3, 4]. One notable condition linked to echogenic kidneys is fetal autosomal recessive polycystic kidney disease (ARPKD), which typically manifests as bilaterally enlarged echogenic kidneys and oligohydramnios on prenatal imaging [5]. Other rare conditions, such as glomerulocystic kidney disease(GCKD), are less frequently prenatally reported [6, 7].

After 16 weeks of gestation, fetal kidneys excrete the fetal urine to the pathway of amniotic circulation, reach the plateau around 34 weeks, steady through the rest of gestation until term, and a little decrease after 40 weeks [8, 9]. The fetal kidneys provide over 90% of the amniotic fluid after 20 weeks and the amniotic fluid acts as a vital sign parameter for fetal well-being [10]. The normality of the amount of amniotic fluid reflects the development and maturity of many organ systems. The fetal swallowing of amniotic fluid is essential for lung maturity, which causes growth and branching of the distal alveolar buds during gestation. Abnormal fetal kidney functions lead to a varying degree of decrease in the amount of amniotic fluid, oligohydramnios, and anhydramnios; this can result in pulmonary hypoplasia and early neonatal death [11]. The management of fetuses with echogenic kidneys and oligohydramnios involves monitoring kidney function through amniotic fluid volume assessment, with decreased or absent fluid indicating a poorer prognosis.

Given the challenging prognosis associated with these conditions, decisions may sometimes be made to terminate the pregnancy, complicating family counseling and limiting opportunities for preimplantation and prenatal genetic diagnosis in subsequent pregnancies. Molecular genetic studies and renal pathology play crucial roles in confirming the diagnosis of a suspected case. In this reported case, we attempt to highlight an alternative differential diagnosis of a cause of fetuses presenting with prenatal ultrasound findings of bilaterally enlarged echogenic kidneys and oligohydramnios.

### Case 1

**Fig. 1:**
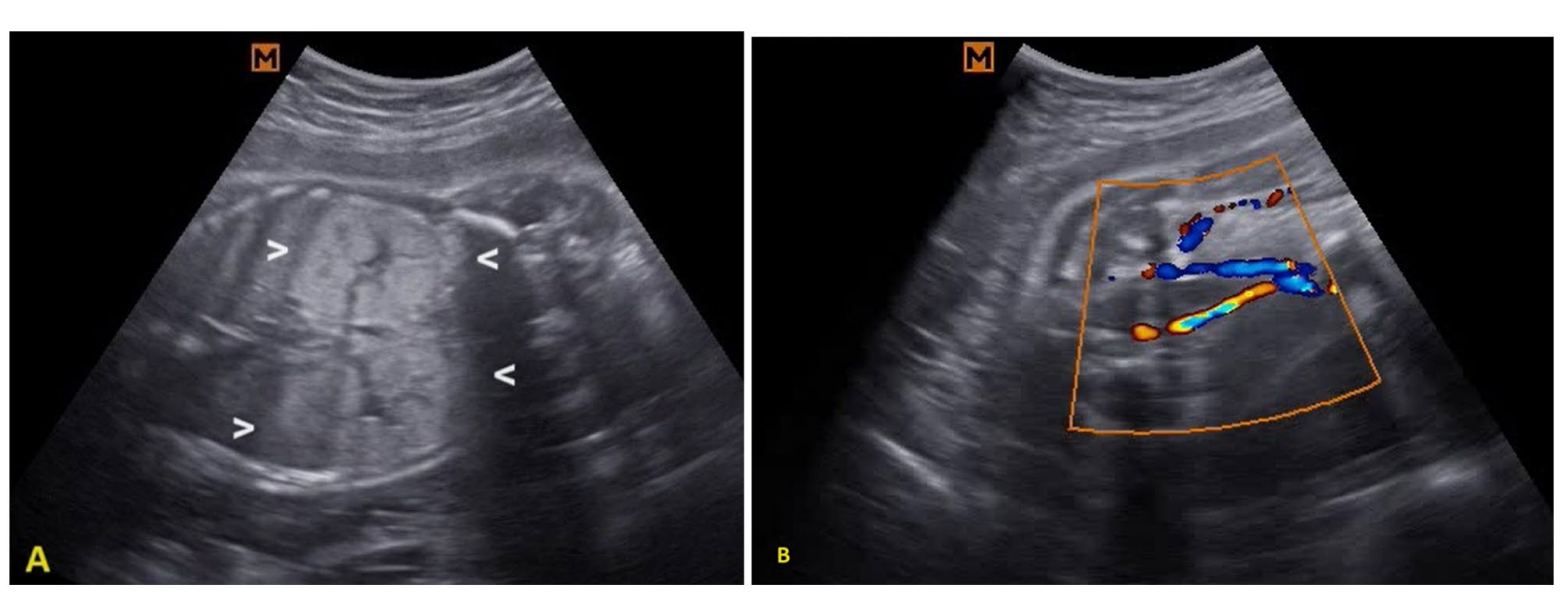
Prenatal 2D grey scale ultrasound. **A** Coronal view of the bilateral enlarged echogenic fetal kidneys with loss of corticomedullary differentiation; **B** Color Doppler ultrasound of umbilical arteries lining the small urinary bladder

**Fig. 2:**
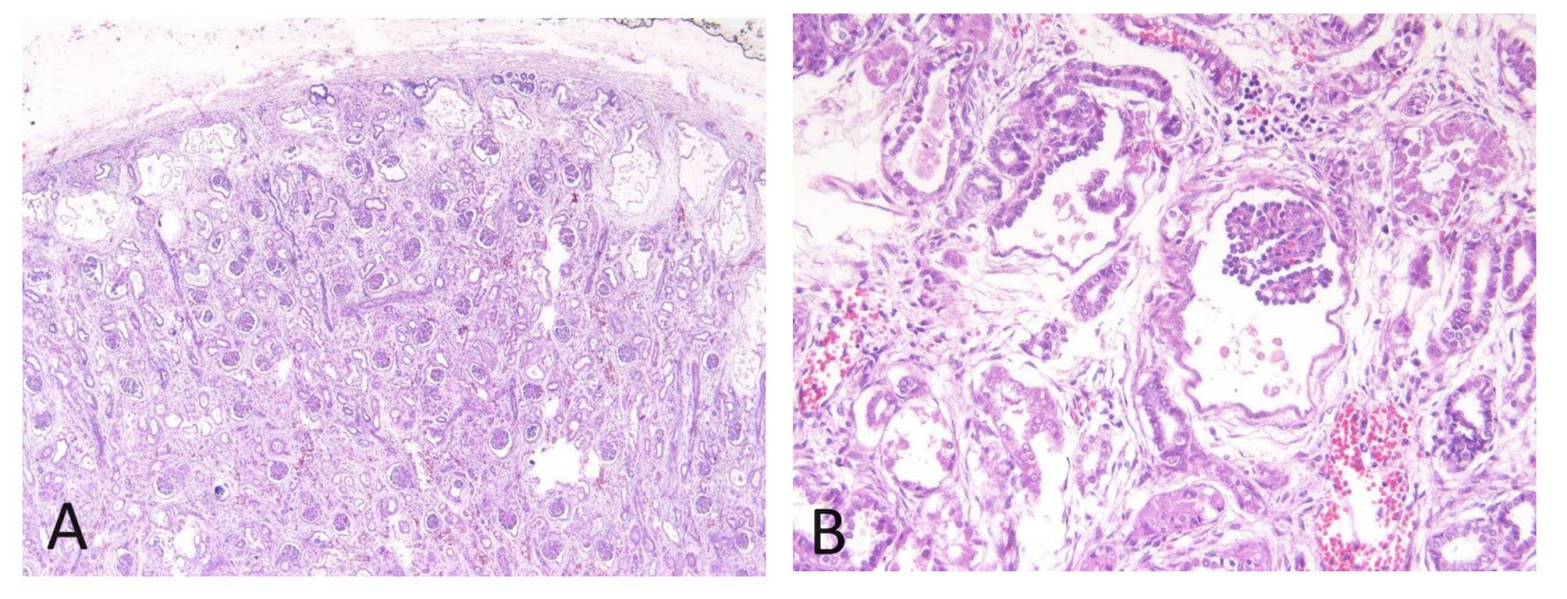
Renal histology revealed dilatation of Bowman’s capsule, leading to the formation of glomerular cysts primarily in the subcapsular cortex. A: H&E stain, 4 × magnification; B: H&E stain, 40 × magnification. Abbreviations: H&E: hematoxylin and eosin

A 43-year-old woman, gravida 2, para 1 (with a healthy 20-year-old daughter), who had underlying essential hypertension, presented with her second episode of antepartum hemorrhage. Family history was unremarkable. Due to an unintended pregnancy, she initiated antenatal care at 25 weeks of gestation at a local primary care unit. Initial antenatal laboratory investigations and management of her hypertension were unremarkable. An ultrasound was performed at 28 weeks and 2 days gestational age due to the antepartum hemorrhage, revealing placenta previa bleeding in the presence of a single viable fetus with appropriate weight for gestational age but also showing signs of oligohydramnios. Following a course of corticosteroids and resolution of vaginal bleeding, she was discharged without immediate fetal concerns. However, ten days later, she returned to the labor room with painless vaginal bleeding. A subsequent ultrasound at 29 weeks and 6 days gestational age showed a viable fetus with an estimated weight of 1,808 g but with anhydramnios and bilateral enlarged hyperechogenic kidneys, the USclip_case1 in supplementary files. The fetal kidneys exhibited abnormal dimensions, with the right kidney measuring 65.4 mm in length (97th centile = 47.7 mm), 36.0 mm in transverse diameter (97th centile = 28.2 mm), and 51.5 mm in antero-posterior diameter (97th centile = 29.2 mm) [12], while the left kidney measured 61.4 mm, 29.9 mm, and 49.8 mm, respectively, showing loss of corticomedullary differentiation (Fig. 1). Color Doppler ultrasound identified a diminutive fetal urinary bladder at the abdominal umbilical cord insertion site. Given the concerning findings and the suspected diagnosis of ARPKD, the patient and her family were counseled about the perinatal poor prognosis. Despite ongoing bleeding from placenta previa, a female neonate was delivered via cesarean section with a birth weight of 1,800 g. The neonate had APGAR scores of 4 and 2 at 1 and 5 min, respectively, and unfortunately passed away 20 min after neonatal resuscitation. A necropsy was conducted, and examination of kidney sections revealed dilatation of Bowman’s capsule, leading to the formation of glomerular cysts primarily in the subcapsular cortex (Fig. 2). These findings were indicative of GCKD, characterized by cyst formation within the glomeruli without significant tubular dilatation or fibrosis in the surrounding stroma. Suspecting cystic kidney disease, genetic testing was initiated. The karyotype analysis revealed 46, XX. Subsequently, singleton whole exome sequencing (WES) with copy number variations (CNVs) analysis was performed using the Illumina HiSeq sequencing system, as detailed in the supplementary material_bioinformatic analysis, to identify potential genetic mutations associated with the condition. Moreover, targeted analysis of 6 genes related to glomerulocystic kidney disease, including *PKHD1*,* PKD2*,* HNF1B*,* TSC1*,* TSC2*, and *NPHP* did not reveal pathogenic/likely pathogenic variants. Unfortunately, the WES and targeted analysis results yielded negative findings, indicating that this specific genetic cause of glomerulocystic kidney disease remained elusive.

### Case 2

**Fig. 3:**
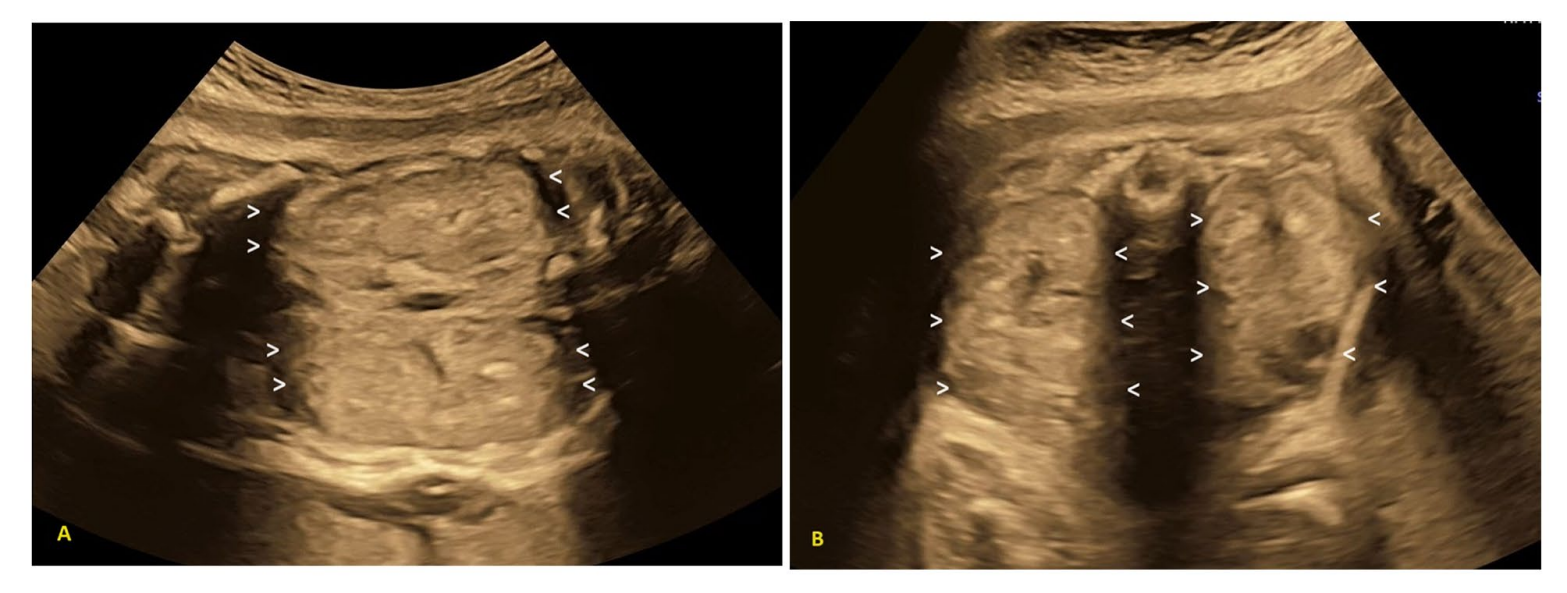
2D grey scale ultrasound of the bilateral enlarged echogenic fetal kidneys with loss of corticomedullary differentiation. The absence of fluid surrounding the fetus was also noted. **A** Coronal view; **B** Axial view

A 30-year-old pregnant woman, gravida 4, para 3002, was referred to the maternal-fetal medicine unit at 29 weeks of gestation due to oligohydramnios. She had no reported underlying medical conditions. Her obstetric history included an early perinatal loss in her second pregnancy, involving a male newborn with severe respiratory distress and abdominal organ enlargement, while her two other daughters were reported as healthy. Antenatal care began at 6 weeks of gestation, with a total of 12 visits leading up to delivery. Initial laboratory investigations during pregnancy were unremarkable. At 31 weeks and 1 day gestational age, a fetal ultrasound revealed a viable fetus with appropriate growth for gestational age, severe oligohydramnios, and abnormal bilateral hyperechogenic enlarged kidneys, the USclip_case2 in supplementary files. The dimensions of the right fetal kidney were notably increased, measuring 56.8 mm in length (97th centile = 46.3 mm), 33.4 mm in transverse diameter (97th centile = 29.90 mm), and 39.1 mm in antero-posterior diameter (97th centile = 29.9 mm) [12], while the left kidney measured 52.8 mm, 29.3 mm, and 31.9 mm, respectively (Fig. 3). A provisional prenatal diagnosis of fetal ARPKD was made. Genetic counseling was provided to the patient and her family regarding the prognosis during the perinatal period. The patient underwent a repeated cesarean section at 35 weeks and 2 days due to spontaneous preterm labor, delivering a preterm female neonate weighing 2500 g with APGAR scores of 8 and 9 at 1-minute and 5-minute intervals, respectively. Unfortunately, the neonate passed away at 9 h of age due to severe respiratory distress, and the parents declined an autopsy. Genetic testing was conducted, revealing a karyotype of 46, XX. Sequence analysis and deletion/duplication testing of a Cystic Kidney Disease gene panel (Invitae, 1400 16th Street, San Francisco, CA 94103, #05D2040778) were performed using the Illumina HiSeq sequencing system, targeting 44 genes associated with cystic kidney diseases as detailed in the supplementary material_bioinformatic analysis. This was followed by Sanger sequencing of the suspected variant, which identified a compound heterozygous likely pathogenic *PKHD1* mutation: specifically, c.3589dup (p.Glu1197Glyfs*42) and c.10105T > C (p.Ser3369Pro). The heterozygous *PKHD1* mutation c.3589dup (p.Glu1197Glyfs*42) introduces a premature stop signal in the *PKHD1* gene and is not present in population databases. Loss-of-function variants in *PKHD1* are recognized as pathogenic, and algorithms designed to predict the effects of sequence changes on RNA splicing suggest that this variant may create or enhance a splice site. In the case of the heterozygous *PKHD1* mutation c.10105T > C (p.Ser3369Pro), this sequence change substitutes serine with proline at codon 3369 of the PKHD1 protein. The serine residue is highly conserved and is also absent from population databases. The segregation of biallelic variants (in trans) of *PKHD1* in the neonate and the heterozygous state in the asymptomatic parents has been confirmed. For these reasons, these variants have been classified as likely pathogenic, confirming the diagnosis of ARPKD in the neonate.

## Discussion

Fetal kidneys are among the structures evaluated during the standard fetal anatomic survey, typically performed by ultrasound at mid-trimester, around 18–22 weeks of gestational age [13]. However, some fetal renal abnormalities may not be detected at this stage because they develop or become more apparent in the late second to third trimester [14]. Approximately 55–60% of fetal urogenital anomalies are diagnosed in the third trimester [15, 16]. This is consistent with the reported cases in which prenatal diagnosis of fetal echogenic kidneys was made at 29 weeks of gestation. Increased renal echogenicity indicates abnormal renal parenchyma, suggesting the presence of multiple microscopic cysts and/or multiple interfaces of dilated renal tubules.

According to several studies, the differential prenatal diagnosis of fetal isolated bilateral enlarged echogenic kidneys involves polycystic kidney diseases, which can be assessed based on amniotic fluid volume. In the absence of urinary tract obstruction, normal amniotic fluid with echogenic kidneys suggests autosomal dominant polycystic kidney disease (ADPKD), whereas decreased or absent amniotic fluid indicates ARPKD [17, 18]. Both conditions are classified as renal ciliopathies. Renal ciliopathies are caused by the defect of primary cilia, the cellular organelles that extend from the surface of many cells, and defects in their structure or function can lead to various cystic phenotypes [19].

Glomerulocystic kidney disease (GCKD) is defined by cystic dilatation of Bowman’s space to more than 2 to 3 times its normal size on histology. GCKD is a rare renal cystic disease with variable classification, diverse etiology, and a spectrum of clinical manifestations [20]. Early-onset GCKD can remain stable for several years or progress to end-stage renal disease within a few years. Regarding pathogenesis, GCKD can be classified as either an isolated kidney disease or part of a genetic syndrome [20, 21]. The pathogenesis of cystic formation in GCKD remains elusive. Several reports suggested that GCKD occurs late in gestation due to dysplasia or tubule obstruction [6, 20]. Our case involved an isolated pathological kidney confirmed without other molecular findings, highlighting that isolated GCKD can present with prenatal findings similar to those of echogenic kidney conditions, particularly ARPKD. A prior review from Cramer MT and Guay-Woodford LM reported that the echogenic kidneys in GCKD can vary in size from small to normal or enlarged [22]. This means that sonographic findings in fetuses with GCKD are difficult to distinguish from those with ARPKD if the enlarged hyperechogenic kidneys and oligohydramnios are the only isolated findings prenatally. In the group of isolated fetal echogenicity with small or normal kidney size, sporadic GCKD should be differentially diagnosed as an underlying cause, especially in negative results of prenatal molecular genetic tests.

GCKD exhibits cyst formation in renal pathology due to dilatation at Bowman’s capsule, whereas ARPKD prominently affects the collecting ducts; however, the prenatal ultrasound cannot differ the location or degree of kidney echogenicity by itself. This distinction can be a crucial clue for differentiating these two syndromes. Not all cases of non-syndromic GCKD have a molecular diagnosis identified. Some reports have indicated the presence of heterozygous *HNF1B* mutations [23, 24], making renal histology the primary method for postnatal diagnosis, which cannot help with the differential diagnosis of imaging findings in the fetus. Reports indicate that prenatal GCKD exhibits a variable clinical spectrum, ranging from severe prognosis to favorable outcomes [6, 7]. In ARPKD, approximately 20-30% of affected infants die during the neonatal period or within the first year of life, primarily due to respiratory insufficiency or secondary pulmonary infections [11]. The prenatally diagnosed hyperechogenic kidney prognosis is closely associated with oligohydramnios, which negatively impacts lung embryogenesis and renal function after birth [25], as demonstrated by the two presented cases. However, the capacity to predict perinatal outcomes remains limited. Advances in neonatal intensive care, including respiratory support and kidney transplantation, have significantly improved survival rates.

Differentiating between these two diseases is essential for precise perinatal management and effective family counseling. In the two reported cases, the prenatal diagnosis was ARPKD, but the postnatal definitive diagnosis differed despite similar prenatal ultrasound findings. This underscores the importance and benefits of postnatal molecular diagnosis and renal pathology for accurate diagnosis and family counseling, including prenatal and preimplantation genetic diagnoses.

## Electronic supplementary material

Below is the link to the electronic supplementary material.

Supplementary Material 1

Supplementary Material 2

Supplementary Material 3

## Data Availability

The datasets generated and/or analyzed during the current study are available in the NCBI repository; accession number to datasets is SAMN4162410 and SRA data is PRJNA1118803. The datasets used, and materials during the current study are available from the corresponding author upon reasonable request.

## Abbreviations

ARPKD: Autosomal recessive polycystic kidney disease
GCKD: Glomerulocystic kidney disease
APGAR: Appearance, pulse, grimace, activity, respiratory
WES: Whole exome sequencing
CNVs: Copy number variations
ADPKD: Autosomal dominant polycystic kidney disease
