# Supplementary material for: A rare cause of echogenic kidneys with oligohydramnios in the fetus: report of two different cases
Source: BMC Pregnancy Childbirth. 2024 Oct 11;24:662. doi: 10.1186/s12884-024-06861-w (PMC11470709; doi:10.1186/s12884-024-06861-w)
Supplement: Supplementary file 3 — Supplementary Material 3 [file 12884_2024_6861_MOESM3_ESM.docx]

**Supplementary material**

**Whole exome sequencing (WES)**

**Variant identification and prioritization**

After informed consent, three milliliters of peripheral blood were collected from the patients. Genomic DNA was extracted from peripheral blood leukocytes using a Puregene blood kit (Qiagen, Hilden, Germany). The DNA samples were then sent to Macrogen Inc. in Seoul, Korea, for exome sequencing. Libraries were enriched using SureSelect Human All Exon V5 kits and sequenced on the Illumina NovoSeq 6000 Sequencer. Sequence reads in FASTQ format were aligned to the Human Reference Genome hg19 from UCSC using Burrows-Wheeler Alignment (BWA) software (http://bio-bwa.sourceforge.net/). Mean depth of coverage was 100 × (> 10× = 99.2%). After aligning the reads to the human reference genome (hg19) with BWA, single nucleotide variants (SNVs) and small insertions/deletions (indels) were identified using GATK HaplotypeCaller and annotated with dbSNP and 1000 Genomes data. Structural variants (SVs) and copy number variants (CNVs) were detected using GRIDSS and ERDS [1, 2]. For variant prioritization, an initial list of 180 genes associated with cystic renal disease was referenced from Genomics England PanelApp (https://panelapp.genomicsengland.co.uk/panels/487/) including targeted analysis of 6 genes ( *PKHD1, PKD2, HNF1B, TSC1, TSC2*, and *NPHP*) related to glomerulocystic kidney disease, focusing first on coding missense, nonsense, frameshift, and splice site variants. Subsequently, Exomiser (https://github.com/exomiser/Exomiser) was employed to identify potential causative variants using HPO terms (HP:0000107; renal cyst, HP:0012210; abnormal renal morphology, and HP:0000077; abnormality of the kidney). If no variants were found within the gene list, candidate pathogenic variants were selected based on the following criteria: (1) population frequency <1% in gnomAD (http://gnomad.broadinstitute.org/) and the Thai reference exome database (T-REx) [3]; (2) variants predicted to impact coding regions (including missense, nonsense, consensus donor/acceptor splice site mutations, and insertions/deletions); and (3) missense variants deemed damaging or disease-causing by at least three in silico predictive mutation impact tools: PolyPhen-2 (http://genetics.bwh.harvard.edu/pph2/), SIFT (http://sift.jcvi.org/), Mutation Taster (http://www.mutationtaster.org/), M-CAP (http://bejerano.stanford.edu/mcap/), and CADD (http://cadd.gs.washington.edu/). Variants were considered novel if they were not reported in the Genome Aggregation Database (gnomAD), ClinVar (https://www.ncbi.nlm.nih.gov/clinvar/), PubMed literature, or identified in our in-house Thai reference exome database (T-REx) [3]. Candidate variants were classified according to the guidelines of the American College of Medical Genetics and Genomics (ACMG), and clinical geneticists reviewed all candidate variants.

**Cystic Kidney Disease gene panel analysis**

Sequence analysis and deletion/duplication testing of a Cystic Kidney Disease gene panel (Invitae, 1400 16th Street, San Francisco, CA 94103, #05D2040778) were performed using the Illumina HiSeq sequencing system, targeting 44 genes associated with cystic kidney diseases including *ALG8, ANKS6, BICC1, CEP164, CEP290, CEP83, CEP89, COL4A1, CRB2, DCDC2, DICER1, DNAJB11, DZIP1L, GANAB, GLIS2, HNF1B, IFT172, INVS, IQCB1, JAG1, LRP5, MAPKBP1, NEK8, NOTCH2, NPHP1, NPHP3, NPHP4, OFD1, PAX2, PKD2, PKHD1, PRKCSH, RPGRIP1L, SDCCAG8, SEC61A1, SEC63, TMEM67, TSC1, TSC2, TTC21B, UMOD, VHL, WDR19, ZNF423.* All targeted regions were sequenced with a depth of ≥50x. Reads were aligned to the reference sequence (GRCh37), and sequence changes were identified and interpreted in the context of a single clinically relevant transcript. The enrichment and analysis focused on the coding sequences of the specified transcripts, including 20 bp of flanking intronic sequences and other specific genomic regions known to be causative of disease at the time of assay design. Promoters, untranslated regions, and other non-coding regions were not analyzed. Exonic deletions and duplications were identified using an in-house algorithm that determines copy number at each target by comparing the read depth for each target in the proband’s sequence with the mean read depth and read depth distribution obtained from a sample set. Markers across the X and Y chromosomes were analyzed for quality control and may detect deviations from the expected sex chromosome complement. Confirmation of the presence and location of reportable variants was performed based on stringent criteria established by Invitae. This was followed by Sanger sequencing of the suspected variant, which identified a compound heterozygous likely pathogenic *PKHD1* mutation: specifically, c.3589dup (p.Glu1197Glyfs*42) and c.10105T>C (p.Ser3369Pro). The heterozygous *PKHD1* mutation c.3589dup (p.Glu1197Glyfs*42) introduces a premature stop signal in the *PKHD1* gene, which is expected to result in an absent or disrupted protein product. This variant is not present in gnomAD population databases. Loss-of-function variants in PKHD1 are recognized as pathogenic, and a reputable source, ClinVar (IDs: 1075810), has recently classified this variant as likely pathogenic. Algorithms designed to predict the effects of sequence changes on RNA splicing suggest that this variant may create or enhance a splice site. In the case of the heterozygous *PKHD1* mutation c.10105T>C (p.Ser3369Pro), this sequence change substitutes serine with proline at codon 3369 of the *PKHD1* protein. The serine residue is highly conserved, and there is a moderate physicochemical difference between serine and proline. This variant is also absent from gnomAD population databases and has been observed in individuals with clinical features of polycystic kidney disease (in-house data). Advanced modeling of protein sequence and biophysical properties—including structural, functional, and spatial information, amino acid conservation, physicochemical variation, residue mobility, and thermodynamic stability—indicates that this missense variant is expected to disrupt PKHD1 protein function. The segregation of biallelic variants (in trans) of *PKHD1* in the neonate, along with the heterozygous state in the asymptomatic parents, has been confirmed. For these reasons, these variants have been classified as likely pathogenic, confirming the diagnosis of ARPKD in the neonate.

**Supplementary reference**

1. Zhu M, Need AC, Han Y, Ge D, Maia JM, Zhu Q, et al. Using ERDS to infer copy-number variants in high-coverage genomes. The American Journal of Human Genetics. 2012;91(3):408-21.

2. Kosugi S, Momozawa Y, Liu X, Terao C, Kubo M, Kamatani Y. Comprehensive evaluation of structural variation detection algorithms for whole genome sequencing. Genome Biol. 2019;20:1-18.

3. Shotelersuk V, Wichadakul D, Ngamphiw C, Srichomthong C, Phokaew C, Wilantho A, et al. The Thai reference exome (T‐REx) variant database. Clin Genet. 2021;100(6):703-12.
